# Effectiveness of Clinical Presentation (CP) Curriculum in teaching clinical medicine to undergraduate medical students: A cross-sectional study.

**DOI:** 10.12688/f1000research.74559.1

**Published:** 2022-02-10

**Authors:** Saroj Adhikari Yadav, Sangeeta Poudel, Swotantra Gautam, Sanjay Kumar Jaiswal, Samikchya Baskota, Aaradhana Adhikari, Binod Duwadi, Nischit Baral, Sanjay Yadav

**Affiliations:** 1 Patan Academy of Health Sciences, Kathmandu, 44600, Nepal; 2 B P Koirala Institute of Health Sciences, Dharan, Nepal; 3 Institute of Medicine, Kathmandu, 44600, Nepal

**Keywords:** Clinical Presentation Curriculum, CP Curriculum, and Medical Education

## Abstract

**Introduction: **The Clinical Presentation (CP) curriculum was first formulated in 1990 at the University of Calgary, Canada. Since then, it has been adopted at various medical schools, including Patan Academy of Health Sciences (PAHS), a state-funded medical school in a low-income country (LIC), Nepal. This study aims to evaluate the perceived effectiveness of the CP curriculum by students and faculty at PAHS, and test knowledge retention through a surprise non-routine exam administered to students.

**Method:** This is a cross-sectional study to evaluate the efficacy of the CP curriculum in teaching clinical medicine to the first batch of MBBS students of PAHS School of Medicine. Ethical approval was obtained from the Institutional Review Committee (IRC)-PAHS (Ref no std1505911069). Perceived effectiveness was evaluated using a set of questionnaires for faculty and students. A total of 33 students and 34 faculty filled the perception questionnaires. Subsequently, a questionnaire consisting of 50 Multiple Choice Questions (MCQs) from different clinical medicine disciplines was administered to test students’ knowledge retention. Out of 49 students, 38 participated in the surprise non-routine exam.

**Result:** A significantly higher number of faculty preferred the CP curriculum compared to the traditional system of teaching clinical medicine (16 vs 11, Kruskal Wallis: 0.023, ie. P-value < 0.05). A significantly higher number of the students liked and recommended CP curriculum in the clinical year of medical education (20 vs. 13 with p-value < 0.05). In the non-routine surprise exam, two thirds of the students scored 60% or above.

**Conclusion:** Both faculty and students perceive that the CP curriculum system is an effective teaching and learning method in medical education, irrespective of their different demographic and positional characteristics. The students’ overall performance was good in surprise, non-routine exams taken without scheduling or reminders.

## Introduction

Sir William Osler, considered the father of modern medicine, emphasized the teacher's role in helping students to observe and reason. He recommended abolishing the traditional lecture method of instruction. ^1^ Medical education is evolving in response to scientific advances and societal needs. ^2^ A well-organized comprehensive knowledge domain has practical implications in clinical problem solving, and appropriate teaching and learning methods play an important role in achieving the educational goals. ^3^

Clinical presentation (CP) is a relatively new and innovative approach to teaching medicine. CP engages medical students in their understanding of the disease process from clinical feature to diagnosis. Students begin studying abnormalities of complaints, examination, and laboratory findings; i.e., signs, symptoms, and laboratory investigations which a patient presents to the doctor with. Students then progress towards diagnosis. The underlying philosophy of the CP Curriculum is that: “The reaction of the human body to an infinite number of insults is always finite and stable over time”. ^4^ For example, if there is any attack on the respiratory system, whether infectious, inflammatory, immunological, traumatic, or iatrogenic; the respiratory system responds through coughing, cyanosis, chest pain, difficulty breathing, noisy breathing, or hemoptysis. ^4^ Thus, the CP Curriculum aims to help students understand the process of moving from “symptoms to diagnosis.”

The CP curriculum was first formulated in 1990 at the University of Calgary Faculty of Medicine in Canada. ^5^ The curriculum was adopted and redesigned based on local needs at various medical schools worldwide. Patan Academy of Health Sciences (PAHS), a state-funded medical school in Nepal, has adopted several new and innovative approaches in teaching and learning medicine. The CP Curriculum is one of the several approaches adopted by PAHS. ^6^

PAHS medical education team assumes that the CP curriculum is better than traditional lecture-based teaching. In this study we are testing the perceived effectiveness of students and faculty, and the level of knowledge among the students trained by the CP curriculum. The level of knowledge was assessed by marks scored by the students in a surprise non-routine exam without prior information. Perceived effectiveness was based on the thinking/perception of the students and faculty on the effectiveness of the CP curriculum. We assume the CP curriculum is at least not inferior to traditional lecture-based teaching.

## Methods

### Study design

This is a cross-sectional study that aims to evaluate the efficacy of the CP curriculum in teaching different disciplines of clinical medicine to undergraduate medical students of PAHS, which is currently the only medical school implementing the CP-curriculum in undergraduate medical education. A new Multiple-Choice Question (MCQ) based questionnaire was designed to evaluate the level of knowledge and two separate questionnaires were developed for faculty to evaluate perception about CP-curriculum.

### Study population

All consenting medical students from 2016 of PAHS School of Medicine currently in clinical clerkship years and all clinical sciences faculty who had delivered at least one teaching-session with the CP curriculum to these students were included in the study. Consenting students were asked to fill the questionnaire together in class, whereas faculty were approached personally and asked to complete the questionnaires. Students and faculty who were part of the study team, those who didn’t provide consent, and those who participated in the pilot survey section of the questionnaire developed for this study were excluded. All 34 faculty completed the perception questionnaires, with zero non-response rate. Out of 49 students, 33 completed the perception questionnaires and 38 turned up to the surprise non-routine exam for assessment of knowledge retention.

### Ethics and consent

This study was approved by the Institutional Review Committee (Ethical Committee) of Patan Academy of Health Sciences (PAHS), Kathmandu, Nepal (Ref No std1505911069). Written informed consent was obtained from all participants before completing the questionnaire. Students who gave verbal consent were asked to complete the questionnaire together in class. Faculty were approached personally and requested to complete the questionnaires. At the start of each questionnaire, a tick box was used for participants to indicate written consent. Participants were informed verbally and in writing that their names and identifiying information would be kept anonymous, and their data would only be used for research purposes.

### Data collection

Three sets of questionnaires were used. The first set of questionnaires were designed to test the perceived effectiveness of the CP curriculum from the faculty perspective. It contained seven questions on background information (age, sex, job position, highest academic degree, etc) and 13 questions on perceived effectiveness.

Similarly, the second set of questionnaires for the students included 11 questions on background information and 15 questions on perceived effectiveness. The perception questionnaire had questions about effectiveness or satisfaction in regard to different aspects of the CP curriculum. Participants had to respond with a tick mark in a Likert scale ranging from one (strongly agree) to five (strongly disagree) for each question.

The third set of questionnaires tested the students' clinical knowledge and contained 50 MCQs from different clinical medicine disciplines. Based on curriculum of the university, there were seven MCQs each from surgery, medicine, pediatrics, obstetrics and gynecology, and two questions each from orthopedics, emergency medicine, general practice, otolaryngology, anesthesiology, dermatology, dentistry, psychiatry, radiology, ophthalmology, and forensic medicine. The questions were randomly selected from the question pool of the Examination section of university. The selected questions were randomly arranged, and a surprise non-routine written exam was conducted with this questionnaire. A maximum time of one hour was provided to solve these 50 questions.

These questionnaires were compiled and discussed in the research group and reviewed by the research advisors to establish content validity. Copies of all three questionnaires can be found under *Extended data.* ^10^ They were administered to randomly selected 15 students and 15 faculty in a pilot study to establish the face validity and feasibility. The students and faculty randomly selected for the pilot study were administered the questionnaires to complete. Then they were asked in detail about the questionnaire and any suggestions for revisions or editing needed. The pilot survey was not powered for statistical comparisons. Only a few grammatical corrections were made after review and feedback from the pilot study. Subsequently, the final study was conducted.

The faculty participants were also involved in the development of the CP curriculum at PAHS, hence, responder bias in favor of CP curriculum may be present in this study.

### Analysis

The data collected were digitalized using Epi-Info version 7 software. These raw data were exported to MS-Excel. The excel sheet is made available in the public domain for readers. ^10^ SPSS version 13.0 was used for statistical test and analysis. Shapiro-Wilk test was used first to test the normality. Non-parametric tests (Mann-Whitney and Kruskal Wallis) was used for normal distribution. Classical ANOVA for equal variance and Welch ANOVA for unequal variance were used after testing the homogeneity of variance, and post-hoc/tukey test was used for significant classical ANOVA results.

## Results

In this study, we calculated the total score via forced Likert scale, ranging from 1 (strongly agree) to 5 (strongly disagree) for each respondent determined as the dependent variable, and compared it with other variables i.e., background information. The total score of all the Likert scale questions was calculated, and the normality test was performed, keeping “total score” as the dependent variable. The full dataset can be found under *Underlying data.* ^10^

### Response from faculty on perceived effectiveness of the CP curriculum

The data of the total score did not follow a normal distribution (Shapiro-Wilk Test, p < 0.05), so a non-parametric test was used to compare the dependent variable. We used Mann-Whitney and Kruskal Wallis tests for the variables containing two groups and more than two groups, respectively.

Among the 34 respondents from the faculty group, 24 (70.59%) were male, and 10 (29.41%) were female. 20 (58.82%) of the faculty respondents were lecturers, and the remaining 14 (41.18%) were senior professors, associate professors, and assistant professors. Out of the 34 respondents, 31 (91.18%) were involved in developing the CP curriculum at PAHS. However, 3 (8.82%) were involved in teaching the curriculum but not in developing the CP curriculum.

As many as 15 (44.12%) respondents favored the CP curriculum system over the traditional system, 11 (32.35%) preferred the traditional teaching system, and 8 (23.53%) preferred both. Overall, the faculty liked the CP curriculum more than the traditional system of teaching clinical medicine (Kruskal Wallis = 0.023, p-value < 0.05). The majority of faculty, 27 (79.41%), would suggest future students to join a medical school that implemented the CP curriculum system rather than the traditional system. Only 12 (35.29%) of them thought that the CP curriculum system should be the sole leading teaching and learning system in clinical medicine, meaning more faculty preferred a hybrid system of both the CP curriculum and the traditional system. However, these differences were not statistically significant (p-value > 0.05).

As shown in Table 1, a significant number of faculty (p values > 0.05) perceive the CP curriculum to be more effective than the traditional system for teaching clinical medicine to undergraduate medical students. There is no significant difference in the perception of the effectiveness of the CP curriculum among faculty based on academic rank, gender, highest academic degree, or the institution of their residency training (p-value > 0.05). The median number of faculty who perceive the CP curriculum system to be more effective and suggest future students to study medicine in this system rather than the traditional system is higher. But, the difference was not statistically significant (p > 0.05). There was no significant difference in faculty foreseeing the CP curriculum as the leading method of teaching and learning medical education in the future (p > 0.05).

**Table 1.** Comparison of demography of the faculty based on forced Likert scale total scores of perception questionnaire for faculty.

Variables	Groups	Total Score				Tests
		Count	Median	Minimum	Maximum	
Position	Professor	4	33	30	40	Kruskal Wallis: 0.444
	Associate Prof.	6	32	10	33	
	Assistant Prof.	4	31.5	30	32	
	Lecturer	20	29.5	19	36	
Gender	Male	24	31	10	40	Mann Whitney: 0.603
	Female	10	31.5	24	36	
Highest degree	MD/MS/MPH	29	32	19	40	Kruskal Wallis: 0.572
	Fellowship	3	29	10	32	
	PHD/DM	2	31	30	32	
Completed residency from	Nepal	22	32	19	40	Mann Whitney: 0.941
	Others	12	30	10	36	
System participants like	CP curriculum	15	32	29	40	Kruskal Wallis: 0.023
	Traditional system	11	25	10	36	
	Both combined	8	29	24	34	
CP System as a leading system	Yes	12	32	29	40	Kruskal Wallis: 0.104
	No	4	25.5	10	32	
	Can't say	18	29.5	22	36	
Suggests to study in medical school implementing CP	Yes	27	32	24	40	Mann Whitney: 0.250
	No	7	29	10	36	
Prefers to teach	CP curriculum	20	32	25	40	Mann Whitney: 0.006
	Traditional system	13	25	10	32	

### Response from students on perceived effectiveness of the CP curriculum

The normality test shows that the total score data follows a normal distribution (Shapiro-Wilk, p > 0.05) with a mean value of 50.57 with a standard deviation of 8.17. Therefore, we used a parametric test to compare the test variable with others. We subsequently tested for homogeneity of variance: we used classical ANOVA for equal variance, and Welch ANOVA for unequal variance. Finally, if significant classical ANOVA results were obtained, we used the post-hoc/tukey test.

There were 33 respondents, among which 23 (69.70%) were males, and 10 (30.30%) were females. The age group of respondents was between 20 to 30 years. A significantly higher number (20 i.e., 60.61%) of the respondents recommended studying in a medical school implementing CP curriculum (p < 0.05). No significant differences were seen between educational or geographical backgrounds and scholarship categories (p > 0.05) as shown in Table 2.

**Table 2.** Comparison between total score and demographic variables of students based on the perception questionnaire for students.

	Result	Remarks
Age	No significant difference (p = 0.161)	
Place of residence	No significant difference (p = 0.298)	Urban Vs Rural
Educational background	No significant difference (p = 0.257)	10+2 Science Vs 10+3 Health science
Pay category	No significant difference (p = 0.161)	Government scholarship vs self funded
Recommendation of CP to future students	Statistically significant difference (p < 0.001)	Mean of perceived effectiveness of students who recommended CP was 54.90 and those who did not was 43.92.

### Assessment for knowledge retention of the students

An hourly surprise non-routine written exam was conducted to test the knowledge of the students. A copy of this exam can be found under *Extended data.* ^10^ The exam included 50 MCQs from different disciplines of clinical medicine. The surprise test was conducted without prior reminders, and 38 out of 49 students participated. The findings, as outlined in Table 3, show that 24 out of 38 (65.79%) of the students scored 60% or higher. The results demonstrate that approximately two-thirds of the students passed the surprise test, indicating good test performance.

**Table 3.** Results of the students’ score in non-routine surprise exam based on the MCQ questionnaires for students.

Percentage range (score)	Frequency	Percentage of students
>80%	4	10.53%
60-80%	21	55.26%
40-60%	8	21.05%
<40%	5	13.16%
	Total = 38	Total = 100%

## Discussion

The current study shows a higher preference for the CP curriculum by undergraduate medical students and faculty at PAHS for teaching and learning clinical medicine in medical school. These findings further substantiate previous reviews on the principles of teaching methods and the acceptability of the curriculum.

This curriculum was chosen in part because of confidence in the comprehensiveness of the knowledge it encompasses. Equally important was the organization of medical knowledge that this curriculum engenders: each clinical presentation is organized according to a variable number of causal diagnostic categories. Each of these categories is identified by a prototype. Exhaustive lists of diagnoses belonging to a given category are avoided. As students' clinical experiences increase and they encounter more diagnoses, the students can add them to the appropriate causal categories stored in their memories. How the diagnostic prototypes are presented allows students to identify the discriminating features within and between each. The process by which students can compare and contrast the distinctive features of each disease is facilitated. It is so because the CP curriculum is well organized and comprehensive. ^3^^,^^7^ Since the CP curriculum is simple to follow and to organize the learning content, students in the CP curriculum also reported less stress due to the volume and complexity of study materials and examinations. ^7^

Prior studies at the University of Calgary demonstrated a substantial effect size on students’ retention of basic science knowledge while participating in the CP curriculum. ^8^ Our study conducted on clinical clerkship year participants showed that two-thirds of students achieved 60% (passing scores) or more in the surprise non-routine exam, signifying a high retention of clinical discipline knowledge. Findings from the current study expand on the effectiveness of the curriculum across medical school years with respect to knowledge retention.

A study done among medical students utilizing the CP curriculum showed a favorable response to the use of schema in the CP curriculum. ^9^ In our study, we could not evaluate the use the schemas of CP to perform clinical assessment in order to reach the appropriate diagnosis. We recommend further studies in this respect. Additionally, long-term knowledge retention was not tested in our study, which could be another important area of investigation.

This study has several other limitations as well. The study was conducted at a single institution, thereby potentially reducing the overall generalizability of the findings. The faculty members recruited as participants for assessing the perceived effectiveness of the curriculum were also involved in the adaptation and development of curriculum at PAHS, hence, potentially increasing responder’s bias in the study by some degree. The cross-sectional nature of the study provides only a limited understanding of the effects of the curriculum over the long term.

## Conclusion

Based on this study, we can conclude that both faculty and students perceive the CP curriculum system as an effective teaching and learning method in medicine, irrespective of their demographic and positional characteristics. The findings suggest higher knowledge retention in knowledge by implementing the CP curriculum during clinical clerkship years. Since the 1990s, CP Curriculum has been established as a multidimensional teaching-learning method in many medical school systems. In the evolving medical education world with rapid digitization, massive turnover of medical and education data, and increased use of remote learning methods, a deeper understanding of influencing variables will help effectively utilize this highly valued curriculum.

## Data availability

### Underlying data

Figshare: CP Curriculum Raw data updated in Excel and PDF. https://doi.org/10.6084/m9.figshare.18666410.v1 ^10^

This project contains the following underlying data:

- Analysis and Raw data.xlsx

### Extended data

This project contains the following extended data:

- CP Questionnaire for Faculties.pdf
- CP Questionnaire for students.pdf
- CP Surprise exam Questionnaire.pdf

Data are available under the terms of the Creative Commons Attribution 4.0 International license (CC-BY 4.0).

## Authors' contributions

SAY, SKJ, AA, and BD conceptualized and designed the study. All 9 authors; SAY, SJ, SP, SG, SB, AA, BD, NB, and SY contributed to data analysis and interpretation. SAY and SP wrote the first draft of the article. All 9 authors, SAY, SP, SG, SJ, SB, AA, BD, NB, and SY critically revised the manuscript and approved the final version of manuscript for publication.
