# Supplementary material for: Effectiveness of Clinical Presentation (CP) Curriculum in teaching clinical medicine to undergraduate medical students: A cross-sectional study
Source: F1000Res. 2022 Feb 10;11:165. [Version 1] doi: 10.12688/f1000research.74559.1 (PMC8968744; doi:10.12688/f1000research.74559.1)
Supplement: Peer review history

# Peer review: Reviewer response for version 1

**DOI:** 10.5256/f1000research.78330.r123303

**Published:** 2022-03-29

**Authors:** Richa Shah

**Affiliations:** 1 Health Action and Research, Kathmandu, Kathmandu, Nepal; 2 International Agency for Research on Cancer, Lyon, France

Dear author(s),

 Thank you for your hard work on this research manuscript. Please find my comments/ queries below.

 The research article deals with the effectiveness of Clinical Presentation (CP) curriculum in teaching clinical medicine to undergraduate medical students. CP curriculum is yet to be adopted in many low- and middle-income countries. The results show that the medical students and the faculty were satisfied with the CP curriculum and believed CP as a stand-alone method of teaching as well as in conjunction with traditional methods of teaching could benefit medical students.

**Study design:**

*“This is a cross-sectional study that aims to evaluate the efficacy of the CP curriculum in teaching different disciplines of clinical medicine to undergraduate medical students of PAHS, which is currently the only medical school implementing the CP-curriculum in undergraduate medical education.”*

- Is PAHS the only medical school implementing the CP-curriculum in Nepal or worldwide?

**Ethics and consent:**

*“Students who gave verbal consent were asked to complete the questionnaire together in class.”*

- Please elaborate on this sentence.
- Was any faculty member present in the class?
- Was the test compulsory or optional?
- Did the students have the right to refuse the test or leave the test in between?

**Data collection:**

- Was the questionnaire in English?
- How long did the questionnaire take to complete?
- How much time were the respondents given to complete the questionnaire?

**Analysis:**

- What were the minimum and maximum possible scores (total or based on questionnaire sets)?

I hope the comments are useful and would enable the author(s) to strengthen this study.

Is the work clearly and accurately presented and does it cite the current literature?

Yes

If applicable, is the statistical analysis and its interpretation appropriate?

Yes

Are all the source data underlying the results available to ensure full reproducibility?

Yes

Is the study design appropriate and is the work technically sound?

Yes

Are the conclusions drawn adequately supported by the results?

Yes

Are sufficient details of methods and analysis provided to allow replication by others?

Partly

Reviewer Expertise:

Global health, gerontology, cancer

I confirm that I have read this submission and believe that I have an appropriate level of expertise to confirm that it is of an acceptable scientific standard, however I have significant reservations, as outlined above.

# Peer review: Reviewer response for version 1

**DOI:** 10.5256/f1000research.78330.r123304

**Published:** 2022-03-18

**Authors:** Jayadevan Sreedharan

**Affiliations:** 1 Department of Community Medicine, Gulf Medical University, Ajman, United Arab Emirates

**Title:** **Effectiveness of Clinical Presentation (CP) Curriculum in teaching clinical medicine to undergraduate medical students: A cross-sectional study.**

 This study aimed to assess the perceived effectiveness of students and faculty and the level of knowledge among the students trained by the CP curriculum. The authors assume the CP curriculum is not inferior to traditional lecture-based teaching.

**Are sufficient details of methods and analysis provided to allow replication by others?:**

 It is not clear why the authors have given MCQ to the faculty (their score is given and statistical test done).

**If applicable, is the statistical analysis and its interpretation appropriate?:**

 The authors mentioned in the article that *"Classical ANOVA for equal variance and Welch ANOVA for unequal variance were used after testing the homogeneity of variance, and post-hoc/Tukey test was used for significant classical ANOVA results",* where they have used this test is not clear in the manuscript.

 The p-value is given in exact value; the importance of p-value is to check whether to accept the null or alternate hypothesis. In the methodology, they mentioned that p-value >0.05 is not significant. Then what more information do the readers get if they include the actual p-value? 

 The sample size of this study is very small and the conclusion from this study can not be generalised to the entire population. 

**Are the conclusions drawn adequately supported by the results?**

 The authors mentioned in the conclusion that *"The findings suggest higher knowledge retention in knowledge by implementing the CP curriculum during clinical clerkship years"*. How the authors reach this conclusion is unclear.

Is the work clearly and accurately presented and does it cite the current literature?

Yes

If applicable, is the statistical analysis and its interpretation appropriate?

Partly

Are all the source data underlying the results available to ensure full reproducibility?

Yes

Is the study design appropriate and is the work technically sound?

Yes

Are the conclusions drawn adequately supported by the results?

Partly

Are sufficient details of methods and analysis provided to allow replication by others?

Partly

Reviewer Expertise:

Epidemiology, Biostatistics, Medical education, Public health

I confirm that I have read this submission and believe that I have an appropriate level of expertise to confirm that it is of an acceptable scientific standard, however I have significant reservations, as outlined above.

# Peer review: Reviewer response for version 1

**DOI:** 10.5256/f1000research.78330.r123301

**Published:** 2022-02-17

**Authors:** Priyanka Panday

**Affiliations:** 1 California Institute of Behavioral Neurosciences & Psychology, Fairfield, CA, USA

This article focuses on the importance of the clinical presentation (CP) curriculum in a particular institute (Patan Academy of Health Sciences (PAHS)) among medical students and faculty in terms of their preference and performance on a surprise non-routine exam. 

- Cross-sectional study is appropriate as a study design for this article.
- Relevant articles from 2020, 2019, and 2004 have been appropriately cited as references.
- The methods used for data collection, as well as the result of the study has been elaborated in detail to ensure accuracy.
- Results are presented in a tabular form and the conclusion derived coincides with the results indicating the effectiveness of the CP curriculum system as an effective teaching and learning method in medicine.
- As far as the statistical analysis is concerned, it is not my area of expertise. However, p< 0.05 for response of effective implementation of the CP curriculum and the response from faculty is statistically significant.

Is the work clearly and accurately presented and does it cite the current literature?

Yes

If applicable, is the statistical analysis and its interpretation appropriate?

I cannot comment. A qualified statistician is required.

Are all the source data underlying the results available to ensure full reproducibility?

Yes

Is the study design appropriate and is the work technically sound?

Yes

Are the conclusions drawn adequately supported by the results?

Yes

Are sufficient details of methods and analysis provided to allow replication by others?

Yes

Reviewer Expertise:

Endocrine disorders, Heart conditions, Medications, COVID-19, Obstetric conditions, Epilepsy, HIV, etc.

I confirm that I have read this submission and believe that I have an appropriate level of expertise to confirm that it is of an acceptable scientific standard.
